# A Web- and Mobile-Based Intervention for Women Treated for Breast Cancer to Manage Chronic Pain and Symptoms Related to Lymphedema: Randomized Clinical Trial Rationale and Protocol

**DOI:** 10.2196/resprot.5104

**Published:** 2016-01-21

**Authors:** Mei Rosemary Fu, Deborah Axelrod, Amber Guth, Joan Scagliola, Kavita Rampertaap, Nardin El-Shammaa, Jason Fletcher, Yan Zhang, Jeanna M Qiu, Freya Schnabel, Karen Hiotis, Yao Wang, Gail D'Eramo Melkus

**Affiliations:** ^1^ College of Nursing New York University New York, NY United States; ^2^ Department of Surgery School of Medicine New York University New York, NY United States; ^3^ NYU Laura and Isaac Perlmutter Cancer Center New York, NY United States; ^4^ Tandon School of Engineering Department of Electrical and Computer Engineering New York University New York, NY United States

**Keywords:** pain, ache, soreness, tenderness, symptoms, lymphedema, breast cancer, health behavior, mHealth

## Abstract

**Background:**

Despite current advances in cancer treatment, many breast cancer survivors still face long-term post-operative challenges as a result of suffering from daily pain and other distressing symptoms related to lymphedema, ie, abnormal accumulation of lymph fluid in the ipsilateral upper limb or body. Grounded in research-driven behavioral strategies, The-Optimal-Lymph-Flow is a unique Web- and mobile-based system focusing on self-care strategies to empower, rather than inhibit, how breast cancer survivors manage daily pain and symptoms. It features a set of safe, feasible, and easily-integrated-into-daily-routine exercises to promote lymph flow and drainage, as well as guidance to maintain an optimal body mass index (BMI).

**Objective:**

To conduct a randomized clinical trial (RCT) to evaluate the efficacy of the Web- and mobile-based The-Optimal-Lymph-Flow system for managing chronic pain and symptoms related to lymphedema. The primary outcome includes pain reduction, and the secondary outcomes focus on symptom relief, limb volume difference by infra-red perometer, BMI, and quality of life (QOL) related to pain. We hypothesize that participants in the intervention group will have improved pain and symptom experiences, limb volume difference, body mass index, and QOL.

**Methods:**

A parallel RCT with a control-experimental, pre- and post-test, repeated-measures design is used in this study. A total of 120 patients will be randomized according to the occurrence of pain. Participants will be recruited face-to-face at the point of care during clinical visits. Participants in the intervention group will receive the Web- and mobile-based The-Optimal-Lymph-Flow intervention and will have access to and learn about the program during the first in-person research visit. Participants in the control group will receive the Web- and mobile-based Arm Precaution program and will have access to and learn about the program during the first in-person research visit. Participants will be encouraged to enhance their learning by accessing the program and following the daily exercises during the study period. Participants will have monthly online self-report of pain and symptoms at 4 and 8 weeks post-intervention. During the two in-person research visits prior to and 12 weeks post-intervention, participants will be measured for limb volume difference, BMI, and complete self-report of pain, symptoms, self-care behaviors, and QOL.

**Results:**

This trial is currently open for recruitment. The anticipated completion date for the study is July 2017. The primary endpoint for the study is absence or reduction of pain reported by the participants at week 12 post-intervention.

**Conclusions:**

The-Optimal-Lymph-Flow is a unique Web- and mobile-based self-care and patient-reported outcome system designed to effectively help women treated for breast cancer manage daily pain and symptoms related to lymphedema. Patients learn self-care strategies from a Web- and mobile-based program and track their symptoms. The RCT will directly benefit all women treated for breast cancer who suffer from or at risk for pain and symptoms related to lymph fluid accumulation.

**Trial Registration:**

Clinicaltrials.gov NCT02462226; https://clinicaltrials.gov/ct2/show/NCT02462226 (Archived by WebCite at http://www.webcitation.org/6du4IupG5)

##  Introduction

### Background

Annually, more than 230,000 women are diagnosed with breast cancer, and currently there are more than 2.9 million breast cancer survivors in the United States [1]. Even years after cancer treatment, about 20-40% of women treated for breast cancer suffer daily from chronic pain and more than 50% of women report multiple distressing symptoms related to lymphedema (ie, the abnormal accumulation of lymph fluid in the ipsilateral upper limb or body) [2-5]. The abnormal accumulation of lymph fluid or lymphedema after breast cancer treatment is a result of obstruction or disruption of the lymphatic system associated with cancer treatment (eg, removal of lymph nodes and/or radiotherapy), influenced by patient personal factors (eg, obesity or higher body mass index [BMI]), and triggered by factors such as infections or trauma [6-8].

Breast cancer survivors without a diagnosis of lymphedema also suffer from pain (40%), tenderness (47.3%), aching (30%), or soreness (32.7%); however, significantly higher number of breast cancer survivors with lymphedema experience pain (45.2%), tenderness (52.4%), aching (61.9%), or soreness (31%) in the ipsilateral upper limb or body [9]. In addition to pain, on average, breast cancer survivors without lymphedema report about 5 distressing symptoms while breast cancer survivors with lymphedema report 10 distressing symptoms related to the accumulation of lymph fluid [9-10]. It is clear that many breast cancer survivors still face long-term post-operative challenges as a result of suffering from daily pain and other distressing symptoms related to lymphedema, despite current advances in cancer treatment.

Pain and symptoms related to the accumulation of lymph fluid following breast cancer treatment remain as the main debilitating late complications that impact the breast cancer survivors’ quality of life [2,3,5,11]. Persistent pain related to cancer treatment is considered a stressful complication since it is perceived as a constant reminder of cancer [2,12] and exerts tremendous limitations on breast cancer survivors’ daily living [2,5]. Pain and other distressing symptoms related to lymphedema following cancer treatment can instigate fears and induce feelings of loss of control [2,3,5]. Specifically, the experience of pain, including tenderness, aching, or soreness, causes significant and unrelenting distress among breast cancer survivors [3]. Such distress is usually heightened when breast cancer survivors expect pain and symptoms related to lymphedema to disappear but instead stay as a “perpetual discomfort” [3]. The negative impact of pain and symptoms related to lymphedema can be a source of considerable disability and psychological distress that negatively influences the patient’s daily living [2,3,11,12], and creates a tremendous burden on the health care system [13]. Nonetheless, in clinical practice pain and symptoms related to lymphedema are still under-recognized and undertreated.

While more research is needed to explore the exact etiology of persistent pain and other symptoms after breast cancer treatment,(eg, arm swelling, breast swelling, chest wall swelling, heaviness, firmness, tightness, stiffness, numbness, burning, stabbing, tingling, and limited limb movement), physiologically, the accumulation of lymph fluid in the affected area or limb may create undue pressure on nerves, producing feelings of pain, aching, tenderness, soreness, burning, tingling, stabbing, and numbness as well as inducing sensations of swelling, heaviness, tightness, and firmness [14-15]. Accumulated lymph fluid in the affected area or limb also leads to stiffness and limited limb movement of arm, shoulder, fingers, and elbow [10,15]. Significant associations are found between pain (including aching and tenderness) and accumulation of lymph fluid in the ipsilateral upper limb [10,15]. Research has also shown that with increased number of symptoms reported, breast cancer survivors’ limb volume increased [10,15]. Limb volume as detected by the infra-red perometer has significantly elevated as breast cancer survivors’ reports of pain, tenderness, aching, swelling, heaviness, firmness, and tightness have increased [10]. On average, breast cancer survivors reported 4 symptoms for those with <5.0% limb volume increase; 5 symptoms for 5.0-9.9% limb volume increase, 7 symptoms for 10.0-14.9% limb volume increase, and 13 symptoms for >15% limb volume increase, respectively (*P*<.001) [10].

Breast cancer survivors are known to have a compromised lymphatic system due to breast surgery, dissection of lymph nodes and vessels, and radiation, which leads to ineffective lymphatic drainage, thus accumulated lymph fluid in the affected area or limb [10,15,16]. In addition to the risk factor of compromised lymphatic drainage from cancer treatment, higher BMI is also an established risk factor for the accumulation of lymph fluid [6-10]. Physiologically, a larger body mass creates a disproportion in lymph transport and capacity, resulting in excess extracellular fluid [6,17]. Women are 1.11 times more at risk for developing lymphedema with every increase of 1kg/m^2^ in their BMI [6-8,16]. Although the known risk factors for symptoms related to accumulation of lymph fluid directly from cancer treatment cannot be avoided, (such as removal of lymph nodes, surgery, radiation, chemotherapy, and hormonal therapy), some risk factors, (such as compromised lymphatic drainage and higher BMI), can be modified through education and self-care strategies [14,18,19].

Patient education focusing on self-care strategies holds great promise for reducing the risk of lymph fluid accumulation [14,18,19]. Research evidence demonstrates that even after controlling for confounding cancer treatment-related risk factors, patient education on self-care strategies remains an important predictor for patient-centered outcomes, including symptom experience and self-care behaviors [14,18,19]. Risk factors, such as compromised lymphatic drainage and higher BMI, can also be modified through self-care strategies [14,19]. Current patient education emphasizes precautionary lifestyle behaviors, such as avoidance of repetitive limb movement, lifting weighted objects, needle punctures, blood draw, and the use of compression garments for air travel in the affected limb [20,21]. To date, there is a paucity of high quality evidence to support these precautionary practices that reduce the risk of lymphedema and relieve pain or symptoms related to lymph fluid accumulation [20,21]. Research is lacking to provide evidence to reduce pain and symptoms related to lymph fluid accumulation through self-care strategies targeting compromised lymphatic drainage and higher BMI.

Grounded in research-driven self-care behavioral strategies [14,19], The-Optimal-Lymph-Flow [22] is a unique patient-centered Web- and mobile-based educational and behavioral program focusing on self-care strategies to lessen the symptom burden by promoting lymph flow and maintaining optimal BMI, targeting compromised lymphatic system and BMI, that is, risk factors for pain and symptoms related to lymph fluid accumulations. Patients learn self-care stratigies through the Web- and mobile-based program which can be downloaded on computer, laptop, as well as any mobile phones and tablets. Its underlying premise is to empower, rather than inhibit, how breast cancer survivors live their lives by emphasizing “what to do,” rather than “what to avoid.” It features a safe, feasible, and easily-integrated-into-daily-routine self-care strategies that include shoulder mobility exercises to promote shoulder function, muscle-tightening breathing, muscle-tightening pumping exercises, and large muscle exercises to promote lymph flow and drainage, as well as general instructions to encourage nutrition-balanced (more vegetables and fruits), portion-appropriate diet (feeling 75% full for each meal), adequate hydration, and sleep to strive for maintaining optimal BMI. Patients can learn and follow all the exercises through avatar video simulations [14,19]. The efficacy of The-Optimal-Lymph-Flow has been demonstrated in our recently published study of 140 patients who received the face-to-face nurse-delivered program [19]. Findings of the study demonstrated that over 90% of patients improved their limb volume at 12-month follow-up. This system has been used successfully for its usability testing. The preliminary usability tested was completed by 30 breast cancer survivors who evaluated the easiness, difficulties, and feasibility of using the system on computer, iPhone, iPad, or other smartphones or tablets. Findings of the usability and feasibility test have demonstrated that patients love the Web-based program, especially the videos using the avatar technology to demonstrate the complicated lymphatic system and illustrate the physiological functions of each exercise and detailed step-by-step instructions for each exercise.

The purpose of the research is to conduct a randomized clinical trial (RCT) to evaluate the efficacy of the Web- and mobile-based The-Optimal-Lymph-Flow system, a patient-centered educational and behavorial symptom management program focusing on promoting lymph flow and optimizing BMI, for managing chronic pain and symptoms related to lymphedema.

### Objectives and Hypotheses

The primary objective of this study is to determine the effectiveness of the Web- and mobile-based The-Optimal-Lymph-Flow system for managing chronic pain, aching, soreness, and tenderness among breast cancer survivors and quality of life related to pain. We hypothesize that participants in the intervention group will have no or less severe pain, aching, soreness, and tenderness, and better quality of life related to pain, aching, soreness, and tenderness in comparison with participants in the control group.

The secondary aim of the study is to evaluate the effectiveness of the Web- and mobile-basedThe-Optimal-Lymph-Flow system for managing symptoms related to lymph fluid accumulation, limb volume differences, and BMI. We hypothesize that participants in the intervention group will have fewer or less severe symptoms related to lymph fluid accumulation, minimal limb volume differences, and better BMI in comparison with participants in the control group.

## Methods

### Design

**Figure 1:**
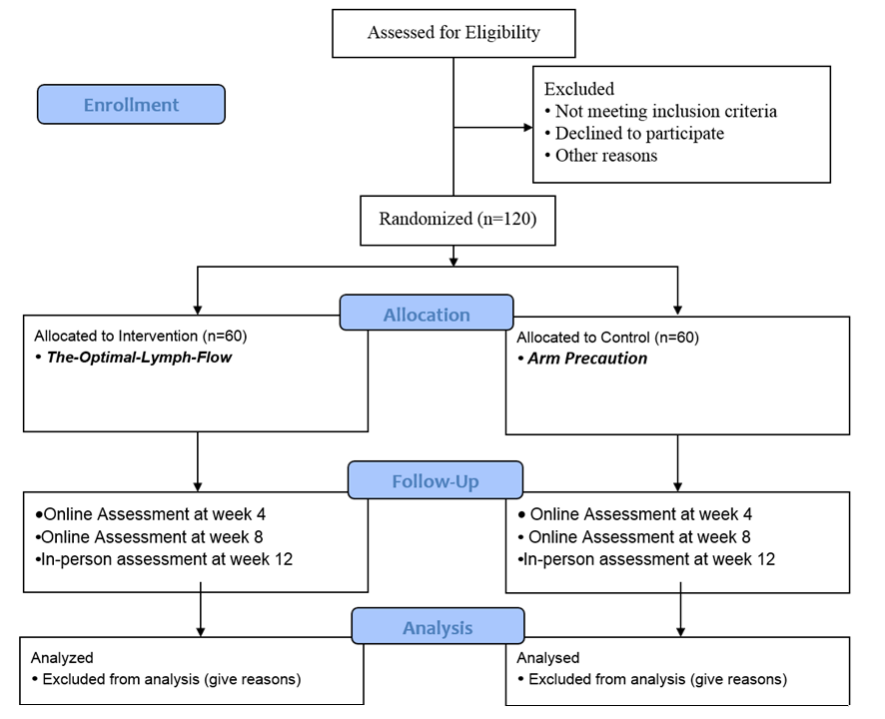
The CONSORT flow diagram.

For this project, chronic pain, including aching, tenderness, soreness, is defined as persistent or intermittent pain in the ipsilateral upper limb or body for more than 3 months after surgical treatment for breast cancer, that is, beyond the expected period of healing [21-23]. A 12-week, two-arm, parallel randomized controlled trial (Clinical trial registration ID: NCT02462226) has been designed to evaluate the effectiveness of the Web- and mobile-based The-Optimal Lymph-Flow self-care strategies to promote lymph flow versus control Arm Precaution group for managing chronic pain and symptoms related to lymphedema. The data collectors will be blinded to the group assignments. The protocol is in accordance with the CONSORT-EHEALTH (see Multimedia Appendix 1) checklist [24]. Multimedia Appendix 2 presents the full proposal review feedback form. Figure 1 shows the CONSORT-EHEALTH flow diagram for recruitment and randomization [24].

### Ethical Approval

This study was approved by the Institutional Review Board of NYU Langone Medical Center on June 8, 2015.

### Study Population

Study population includes: (1) patients who have been surgically treated for breast cancer more than 3 months (healing usually occurs within 3 months of surgical treatment for cancer); (2) patients who report *persistent or intermittent* pain, including aching, tenderness, soreness; (3) patients may or may not report any of the symptoms related to lymphedema (ie, swelling, heaviness, tightness, firmness, numbness, tingling, stiffness, limb fatigue, limb weakness, and impaired limb mobility of shoulder, arm, elbow, wrist, and fingers); (4) patients may or may not have a history of lymphedema or have been treated for lymphedema; (5) patients have Internet access to the Web- and mobile-based program at home or willing to access the program using the laptop provided by the researchers at the cancer center; (6) ability to understand and the willingness to sign a written informed consent document.

Exclusion criteria are (1) patients who do not report any pain, including aching, tenderness, and soreness; (2) patients who have known metastatic disease or other bulk disease in the thoracic or cervical regions; (3) patients who have lymphedema due to cancer recurrence; (4) patients with documented advanced cardiac or renal disease.

### Recruitment

#### Recruitment Process

Participants have been recruited face-to-face at point of care during clinical visits from New York University (NYU) Cancer Center. To accomplish recruitment of 120 participants, we plan to use the successful procedures of recruiting and consenting participants used by the PI and the team in the preliminary studies [3,9,14,17,19]. Successful strategies include the use of *Invitation Flyer* that describes the study. This *Invitation Flyer* is posted on the bulletin boards or breast cancer support website at the cancer center, and is also available in the reception areas of the cancer center, examination rooms, and rooms holding support group meetings. In addition, health care providers such as nurses, oncologists, breast surgeons, and oncology radiologists at the center are willing to refer women meeting the inclusion criteria to the study by distributing the *Invitation Flyer* that describes the study to the potential participants.

#### Consent Process

After reading the *Invitation Flyer*, if a woman is interested in participating in the study, she would schedule a meeting with the research coordinator at that time or at other convenient time for them. During the meeting, the research coordinator will confirm her interest, determine if the woman is eligible for the study and the research coordinator will again explain the study in detail and provide enough time for the woman to ask questions. If the woman agrees to participate, she will sign the consent form.

#### Confidentiality

Confidentiality will be maintained. Patients will be assigned a study ID specific for the study. Study data recording for the research will only use the study ID without the patients’ identifying information. A document file that has the patients’ study ID with the patients’ identifying information will be separately stored in locked files accessible only to the research coordinator, research nurse, or the PI. Electronic data will be stored in a password protected computer accessible only to the PI, the research nurse, and the research coordinator. Data analysis will be carried out and reported in the aggregate data so that individual identities are not revealed. Careful training and supervision of research coordinator and research nurse will insure study procedures are carried out in accordance with established protocols.

### Randomization and Blinding

The randomization assignment will be generated by our senior statistician using a computer-generated randomization procedure. Participants will be randomized based on their report of pain/aching/soreness or tenderness to be allocated to intervention and control group. The researchers who perform pre- and post-intervention measurements will be blinded throughout the study to the participants’ assigned arm. Participants will not know which intervention was the intervention of interest and which one was the comparator.

### Study Intervention

#### Overview

**Figure 2:**
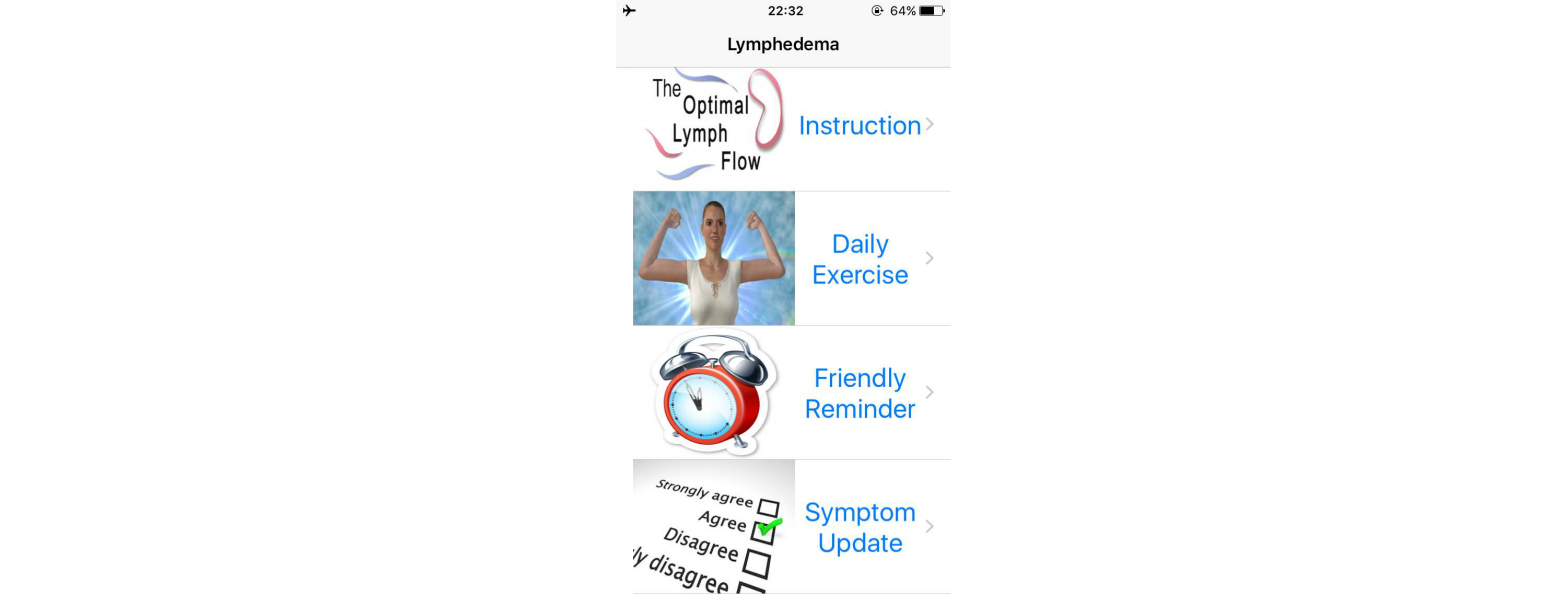
The Web- and mobile-based The-Optimal Lymph-Flow.

**Figure 3:**
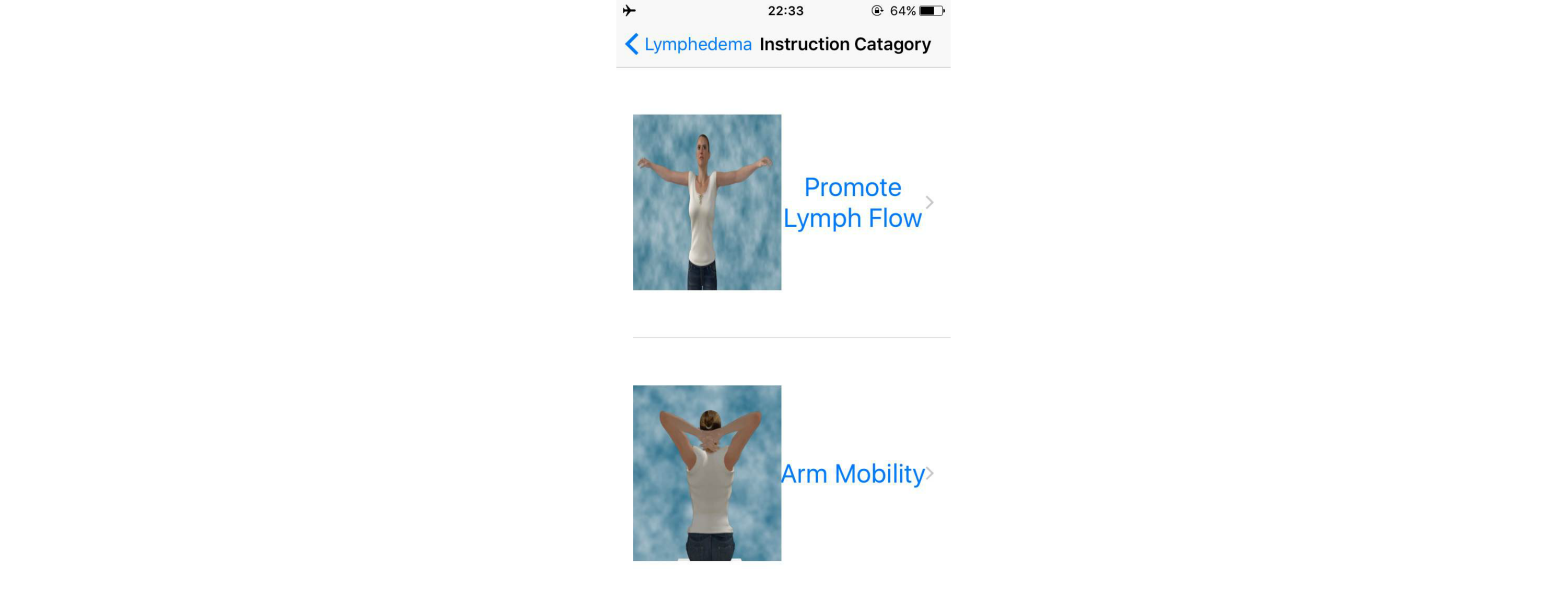
Video instructions for daily exercises.

**Figure 4:**
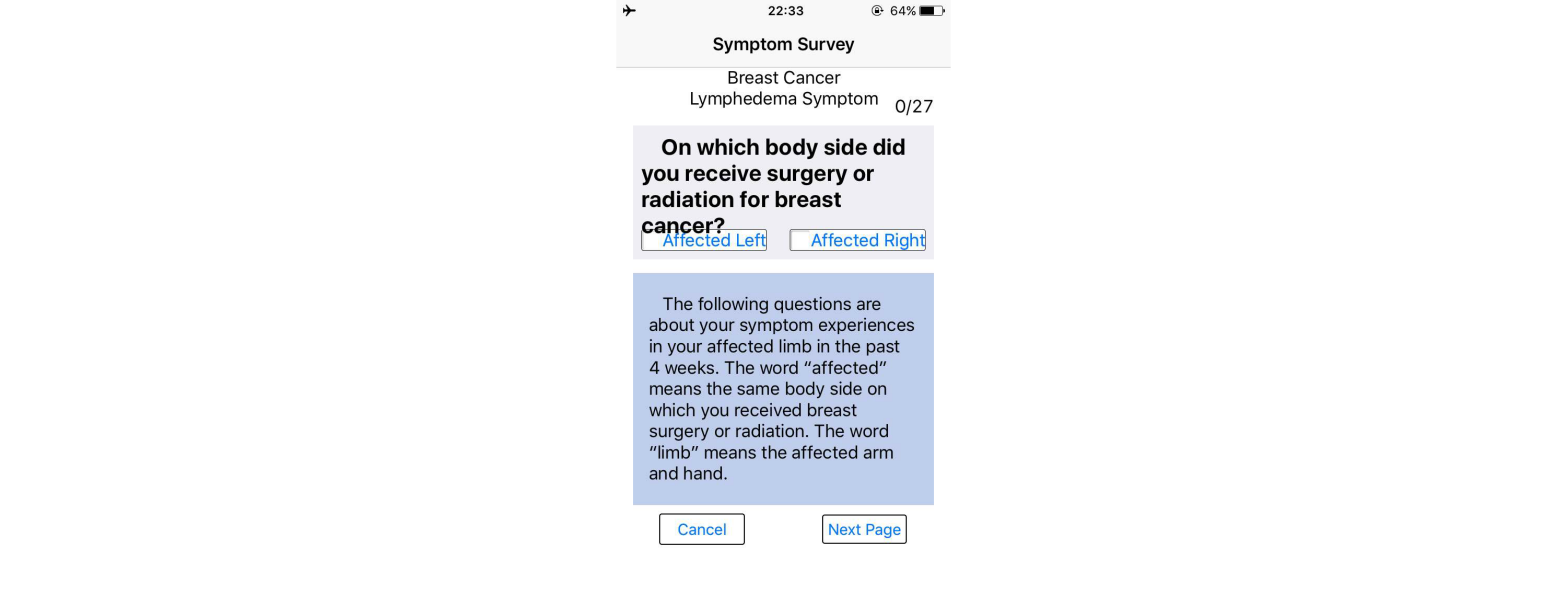
Symptom reporting.

**Figure 5:**
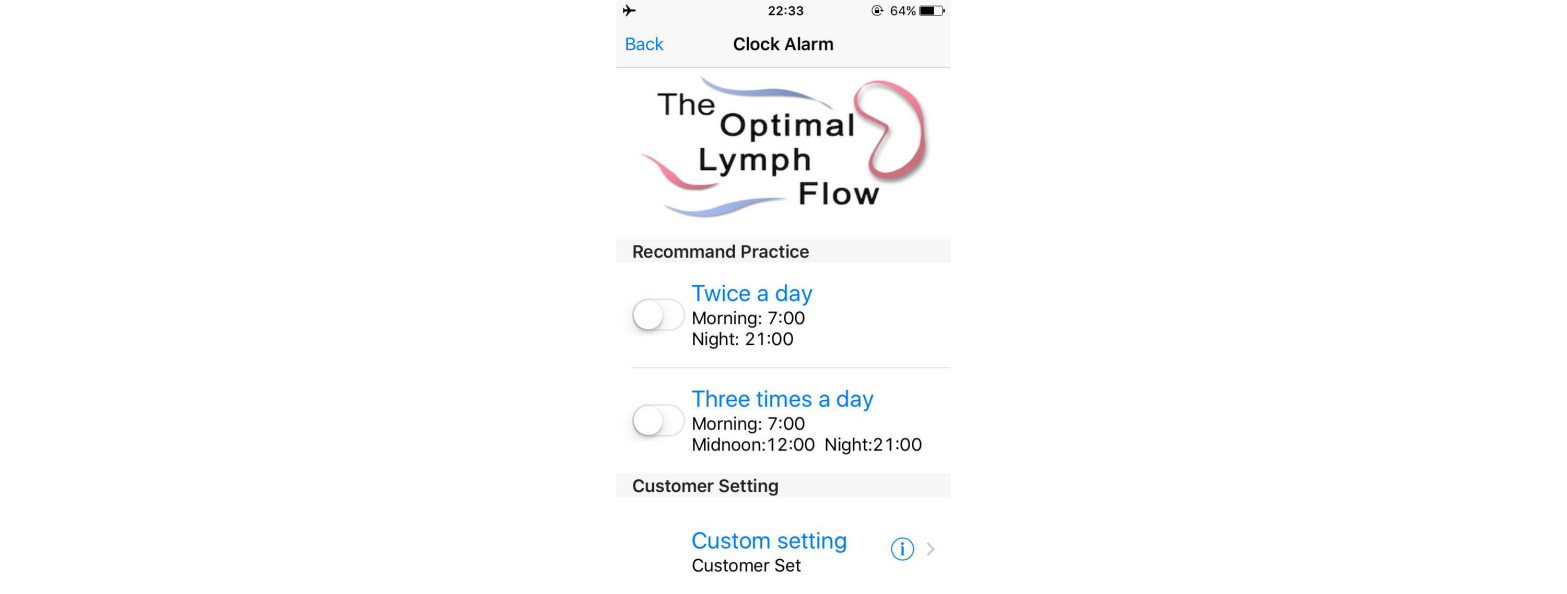
Friendly reminder for daily exercises.

The Web- and mobile-based *The-Optimal-Lymph-Flow* [19,22] includes information about lymphedema, diagnosis and measurement of lymphedema, lymphatic system, risk of lymphedema, self-care, daily exercises, arm precautions, and ask experts. Participants in the intervention group will have access to the 8 Avatar videos that provide step-by-step instructions for The-Optimal-Lymph-Flow exercises to promote lymph flow and optimize shoulder and limb mobility. The platform also has a section entitled Arm Precautions, representing current patient education that emphasizes precautionary lifestyle behaviors, such as avoidance of repetitive limb movement, lifting weighted objects, needle punctures, blood draw, and the use of compression garments for air travel in the affected limb [20,21]. Figures 2-5 shows some screenshots of the The-Optimal-Lymph-Flow program. Table 1 presents the strategies, rationales, and actions for *The-Optimal-Lymph-Flow* program.

**Table 1:** The-Optimal-Lymph-Flow program: self-care strategies, rationales, and actions

Strategies		Rationales	Actions
**Promoting lymph flow**			
	Muscle-tightening deep breathing	The whole body lymph fluid has to be drained through the lymphatic ducts above the heart. Muscle-tightening deep breathing stimulates lymphatic ducts and help lymph fluid drain.	At least twice a day in the morning & at night before brushing teeth or as much as the patient wants throughout the day.
		Lymph fluid drains when muscles move. Muscle-tightening deep breathing creates the whole body muscle movements that create muscle milking and pumping action and help to drain lymph fluid.	Air-travel: before take-off and after landing.
			Sedentary lifestyle: at least every 4 hours.
	Muscle-tightening pumping	Muscle-tightening pumping exercises create arm muscle pumping. This helps lymph fluid flow and decreases the fluid build-up in the arms.	At least twice a day in the morning & at night before brushing teeth or as much as the patient wants throughout the day.
		Muscle-tightening pumping exercises build the arm muscle that helps lymph fluid flow and drain.	Air-travel: before take-off and after landing.
			Sedentary lifestyle: at least every 4 hours.
	Large muscle exercises: walking, marching at home, dancing, swimming, Yoga, Tai Chi	Large muscle exercises create muscle milking and pumping to promote overall body lymph fluid flow and drain.	At least 10-minutes daily.
			Air-travel: get up and walk around for flight over 4 hours.
			Sedentary lifestyle: get up and walk at least every 4 hours.
**Improving limb functional status**			
	Shoulder exercises	Improved limb mobility after surgery facilitates local muscle movements that create muscle milking and pumping to promote local limb lymph fluid flow and drain.	One week after surgery if there is no surgical drains or after the surgical drains are removed.
			At least twice a day until limb functions are returned to normal.
			Whenever limb mobility is limited throughout the recovery.
**Keep a healthy weight**			
	Eat nutrition-balanced diet (ie, more vegetables and fruit as well as quality proteins); Maintain portion-appropriate diet (feeling 75% full for each meal)	Overweight or obesity is an important risk factor for lymph fluid accumulation.	Each meal daily
		Having extra weight makes it difficult for lymph flow and drain. This can lead to extra lymph fluid build-up.	It is important to talk to the nutritionist who can help to find proper weight reduction programs.
		There are numerous weight management programs available to assist with weight loss.	
		Although there are a lot of weight reduction programs, each person may respond differently to each program.	
		The core of the weight management is to eat a nutrition-balanced, portion-appropriate diet. It is also important to stay hydrated, exercise, and get adequate sleep.	
	Stay hydrated	People may actually be thirsty, not hungry.	Drink 6 to 8 glasses of water daily; in the morning, before and during meals, and throughout the day.
			Avoid drinks with calories (eg, juices).
			Drink green tea to boost metabolism.
	Large muscle exercises	Daily large muscle exercises (eg, walking, running, swimming, Yoga) help to burn more calories.	At least 30-minutes 3 times a week or daily.
		Daily large muscle exercises also promote lymph flow by creating muscle pumps.	
	Get enough sleep	Lack of sleep increases the production of the stress hormone cortisol, creates hunger, and leads to overeating.	At least 7-8 hours of sleep per night.
		Getting just one more hour of sleep per night reduces belly fat accumulation.	

#### The-Optimal-Lymph-Flow Intervention Group (n=60)

Patients assigned to The-Optimal-Lymph-Flow intervention group will have access to the Web- and mobile-based The-Optimal-Lymph-Flow platform and they will learn about theprogram and daily exercises during the first in-person research visit. Patients will have access to the website contents of Lymphedema, Diagnosis of Lymphedema, Lymphatic System, Self-care, Daily Exercises, and Ask Experts. Patients will also have access to the 8 avatar videos that provide step-by-step instructions of daily exercises to promote lymph flow and optimize shoulder and limb mobility. In addition, the patients will be introduced to an app, and have the choice to use either the Web-based program or the app for daily exercises. However, patients *will not* have access to the section Arm Precautions since the participants in the intervention group will receive comparable information as in the Arm Precautions section, but with particular emphasis on “what to do,” rather than “what to avoid.”

#### Control Arm Precaution Group (n=60)

Patients assigned to the control Arm Precaution group will have access to the website section that emphasizes on precautionary lifestyle behaviors, such as avoidance of repetitive limb movement, lifting weighted objects, needle punctures, blood draw, and the use of compression garments for air travel in the affected limb [20,21]. Patients will have access to the following contents of the website: Lymphedema, Diagnosis of Lymphedema, Risk of Lymphedema, Lymphatic System, 3 avatar videos for Daily Exercises to promote limb mobility, and Arm Precautions. However, patients will not have access to The-Optimal-Lymph-Flow program, including Self-care*,* Daily Exercises to promote lymph flow, and 8 Avatar videos as well as Ask Experts that allows them to email to the researchers about their self-care. Patients will access to the Web- and mobile-based Arm Precaution program and learn about the program and daily exercises to promote limb mobility during the first in-person research visit.

#### Duration of Intervention

From the previous one-arm clinical trial and usability and feasibility studies on the Web- and mobile-based The-Optimal-Lymph-Flow [19,23], we estimate that it would take about 45-60 minutes for patients to learn all the sections of the program and about 15 minutes to learn the The-Optimal-Lymph-Flow exercises for the intervention group through 8 avatar videos. It takes about 5 minutes to perform a set of The-Optimal-Lymph-Flow daily exercises each time. We encourage patients in the intervention group to perform at least twice a day or more times of the exercises during the study period. Participants in the control group will have access to three limb mobility exercise avatar videos and it takes less than 3 minutes to perform a set of limb mobility exercises each time. Patients in the control group will be instructed to perform limb mobility exercises at least twice a day or more times during the study period.

### Data Collection

#### Data Collection Procedures

Data will be collected at baseline prior to intervention, and at week 12 post-intervention. Data collection at each time point will take approximately 30 minutes. Within one week of enrollment for the clinical trial, patients will have baseline assessment of pain and symptoms, limb volume difference, BMI, and quality of life. The follow-up assessment will occur at week 12 post intervention.

*Two In-Person Research Visits*: (1) prior to intervention there will be baseline assessment of pain and symptoms, limb volume difference, BMI, and quality of life; and (2) 12 weeks post-intervention assessment of pain and symptoms, limb volume difference, BMI, self-care behaviors, and quality of life.

*Two Online Assessments:* patients in the intervention and control group will receive an email that provides a link to assess pain and symptoms as well as quality of life at week 4 and week 8 post-intervention. Confidentiality of the patients will be protected for the online assessment since patients will use their study ID to access the online assessment.

#### Outcome Measures

*Demographic and Medical Information*: a structured tool is used to gather demographic and medical information and is verified through reviewing participants’ medical records [14,17,19]. The demographic and medical information will be considered as covariates, including expectation of the program, pain medications, age, surgeries, lymph nodes procedure, radiation, chemotherapy, time since surgery, time since lymphedema diagnosis, and hormonal therapy.

*Primary and Secondary Outcome Measures*: primary measure focuses on pain which is assessed during prior to the intervention and week 12 post-intervention in-person visit, as well as week 4 and 8 post-intervention online assessment. Secondary measures include symptoms, limb volume difference by infra-red perometer, BMI, quality of life related to pain. Limb volume difference by infra-red perometer and BMI are only measured prior to and week 12 post-intervention in-person visits. Symptoms and quality of life related to pain are assessed prior to and week 12 post-intervention in-person visit as well as week 4 and 8 post-intervention online assessment.

*Pain and Symptoms Related to Lymphedema:* the Lymphedema and Breast Cancer Symptom Experience Index is a valid and reliable self-report tool to assess pain, including aching, soreness, tenderness, as well as symptoms related to lymphedema (ie, arm swelling, breast swelling, chest wall swelling, heaviness, firmness, tightness, stiffness, burning, stabbing numbness, tenderness, stiffness, redness, blistering, and tingling (pins and needles) [14,17,19]. Each symptom can be treated as categorical variable by choosing a “Yes” or “No” to indicate the presence or absence of a given symptom. Each item can also be rated on a Likert-type scale from 0 (no presence of a given symptom) to 4 (greatest severity of a given symptom). Higher scores indicate more severe symptom presence. A response frame of last three months will be used for all participants to ensure the chronicity of symptom presence during the first in-person visit.

*Limb Volume Difference by Infra-Red Perometer*: perometry 350S will be performed on each arm as it is held horizontally. The perometer maps a 3-dimensional graph of the affected and non-affected extremities using numerous rectilinear light beams, and interfaces with a computer for data analysis and storage. A 3-dimensional limb image will be generated and limb volume will be calculated. This optoelectronic method has a standard deviation of 8.9 ml (arm), less than 0.5% of limb volume with repeated measuring [17,19] .

*Quality of life Related to Pain*: the Pain Impact Questionnaire (PIQ-6), a reliable and valid six question health survey, will be used to measure pain severity and the impact of pain on an individual's functional health and well-being. The PIQ-6 measures the severity of pain and its impact on work and leisure activities, as well as on emotional well-being within a variety of diseases and general populations. High PIQ-6 *T* scores indicate greater pain impact/worse health [21-23].

*Height, Body Weight, and BMI*: height will be measured to the nearest 0.1 cm with a portable stadiometer (Scale-Tronix 5002 Stand on Scale, Scale-Tronix Company, Carol Stream, IL, USA) without shoes [25]. An electrical device (InBody 520, Biospace Co, Ltd, Seoul, Korea) will be used to measure the participants’ body weight, BMI is calculated using the formula: weight (kg)/height (m^2^) [25].

*Practice of Self-Care Behaviors:* Risk Reduction Behavior Checklist is a structured self-report checklist that will be used to quantitatively and qualitatively assess patients’ practice of self-care behaviors at the study endpoint of 12-week after intervention [17,19]. The checklist include a list of self-care behaviors that promote lymph flow, eg, muscle-tightening deep breathing, muscle-tightening pumping, shoulder exercises, large muscle exercises, and having nutrition-balance and portion-appropriate diet, adequate hydration and sleep, as well as compression therapy for lymphedema.

### Statistical Analysis

#### Primary Endpoint

The primary endpoint for the study is absence of pain or pain reduction reported by the participants at week 12 post-intervention.

#### Sample Size and Power Calculations

We will enroll a total of 120 participants: 60 participants in The-Optimal-Lymph-Flow intervention and 60 participants in the Arm Precaution control group to account for a potential attrition of 20%, which has been observed in the prior studies in breast cancer survivors [10]. This will yield an adequate analytic sample size. Even with 20% attrition based on a 2 sample 2-sided *t* test with alpha=.05 and power of 90%, we can detect a difference of 0.7 standard deviations in the difference between the presence of pain in the intervention group compared to the control group at 8 weeks or at 12 weeks. The projected sample size will also provide sufficient statistical power for mixed regression models. For linear mixed models of continuous outcomes (eg, pain ratings), statistical power will exceed 80% to detect a medium effect, assuming a constant group effect, correlations of r=.5 between observations, alpha=.05, and compound symmetry of the covariance structure.

For binary outcomes, based on the three repeated observations with a conservative estimate for the assumed correlation of r=0.5 between observations, alpha=.05, sample size of n=50 per treatment arm will have power of 80% to detect odds ratios of the difference between groups ranging from 2.6 to 3.5 (small to medium effects).

Adequacy of sample size will be monitored during the analyses in three ways: (1) assessing sufficiency to estimate the number of model parameters identified by preliminary analyses; (2) examining model fit indices (eg, intra-class correlation coefficient to assess fit of random effects) to ensure data adequately support models generated, and (3) assessing the adequacy to accurately estimate model parameters by generating confidence intervals.

#### Analysis Plan

We will summarize graphically and numerically the distributions of pain, aching, soreness, tenderness, other symptoms related to lymph fluid accumulation, limb volume differences, BMI, and quality of life as well as covariates (such as medication for pain, age, or education and treatment variable and limb volume and self-care behaviors). The proportion of individuals experiencing pain, aching, soreness, tenderness will be compared between the intervention and control group over time, as will the mean severity ratings and associated quality of life scores. Mean comparisons of number of reported symptoms, limb volume, and BMI will be conducted between the intervention and control group as well as over time.

Bivariate relationships between the variables and treatment group will be assessed using point-biserial correlations and phi coefficients to identify potential covariates for inclusion into statistical models. Linear mixed effects models will be used to analyze continuous outcomes (eg, ratings pain, aching, soreness, tenderness, symptom ratings, and quality of life) and generalized linear mixed models will be used to analyze binary outcomes (presence of pain, aching, soreness, tenderness, and total scores of quality of life). These models will incorporate fixed effects for time, group, and any identified covariates. As indicated by preliminary models to estimate variance effects, models will include random effects for subject-specific slopes and intercepts. A statistically significant fixed effect for group by time interaction will indicate a treatment effect, with the direction of difference determined from mean values. Models will be compared for goodness of fit and modifications to link functions, distributional form, and correlation structure will be made as necessary.

#### Interim Analyses

No interim analysis will be conducted since the intervention is only 12-week long.

#### Method of Handling Missing Data and Non-Adherence to Protocol

Data from participants who are missing >20% of any scale will be excluded from calculation of that scale, though remaining data meeting requirements for completeness will be retained. Analysis of missing data will first determine whether it can be assumed to be missing at random (MAR) or not missing at random (NMAR). Based on the results of this step, appropriate methods will be selected for addressing missing data (eg, Heckman Selection for NMAR, Multiple Imputation for data which are MAR).

Further, mixed effects regression models are robust in the presence of missing data. Unlike traditional repeated measures designs (eg, repeated measures ANOVA) which employ listwise deletion, excluding all records for individuals who miss a single observation, mixed effects models are capable of incorporating all completed observations for estimations. Although our previous studies suggest that we will have a limited number of missed observations, individuals need not have the same number of observations. This maximizes the statistical power of our analyses and reduces the likelihood of systematic bias in estimates.

## Results

This trial is currently open for recruitment. The anticipated completion date for the study is July 2017. The primary endpoint for the study is absence or reduction of pain reported by the participants at week 12 post-intervention.

##  Discussion

### Hypothesis

Low cost and pragmatic self-care strategies for symptom management may hold great promise for improving patients’ quality of life [6,19]. To date, limited research has been designed to help breast cancer survivors to manage their daily distressful symptoms, including pain. This clinical trial focuses on primary outcomes of pain reduction and secondary outcomes of relief of symptoms related to lymphedema, limb volume difference by infra-red perometer, BMI, quality of life to refine procedures and estimate effect size for the future efficacy of multi-center RCT. We hypothesize that participants in the intervention group will experience no or less severe pain, aching, soreness, and tenderness and better associated quality of life (related to pain, aching, soreness, and tenderness) in comparison with participants in the control group when compared to participants in the control Arm Precaution group.

### Conclusions

The proposed project will directly benefit all women treated for breast cancer who suffer from or are at risk for pain and symptoms related to lymph fluid accumulation by providing a low-cost, technologically-driven delivery model to universally expand the accessibility of The-Optimal Lymph-Flow. With health care reform under way, using Web- and mobile-based technology to develop low cost and pragmatic patient-centered intervention is the key to lessening the health care cost and advancing the science of symptom management.

## Appendix

Multimedia Appendix 1 CONSORT-EHEALTH checklist V1.6.1.

Multimedia Appendix 2 Full proposal review feedback form.

## Abbreviations

BMI: body mass index
MAR: missing at random
NMAR: not missing at random
PIQ-6: Pain Impact Questionnaire-6
RCT: randomized controlled trial
