# Supplementary material for: A Web- and Mobile-Based Intervention for Women Treated for Breast Cancer to Manage Chronic Pain and Symptoms Related to Lymphedema: Randomized Clinical Trial Rationale and Protocol
Source: JMIR Res Protoc. 2016 Jan 21;5(1):e7. doi: 10.2196/resprot.5104 (PMC4742618; doi:10.2196/resprot.5104)
Supplement: Multimedia Appendix 2 [file medinform_v5i1e7_app2.pdf]

New York University

The Optimal Lymph-Flow ™

Grant ID # 13371953

	Maximum Score	Evaluation Criteria Guidelines	Avg. Reviewer Score for this Proposal (%)
**Overall Goal Alignment**	7%	The overall goal for this project was described in terms of SMART goals (specific, measurable, attainable, relevant and time-bound) and is in line with the specific area of interest for this RFP.	5%
**Project Objectives**	7%	Project objectives are SMART and are in line with the overall goal of the project.	5.6%
**Assessment of Need**	15%	Quantitative baseline data, initial metrics (e.g., quality measures), or a project starting point (gap analyses or patient-level data describing the problem) are included. Baseline data collection and analyses methods are clearly described.	10%
**Target Audience Alignment**	10%	The primary audience(s) targeted to benefit from this project is clearly described and appropriate.	6.4%
**Project Design and Methods**	25%	The study methodology is clearly described, well designed and adequate to achieve the project objectives.	20%
**Outcomes**	20%	Data collection approaches, proposed statistical tools and analyses, and methods to control for bias are clearly described and are appropriate to the project objectives.	16%
**Change Expectation**	3%	The expected impact of the study project on the target audience is clearly stated (e.g., a 10% increase over baseline or a decrease in utilization from baseline between 20-40%).	2.2%
**Audience Engagement**	3%	A description of how the study will determine if the target audience was fully engaged in the project is included.	2.4%
**Dissemination of Project Outcomes**	5%	A plan for broadly disseminating the project outcomes is clearly articulated.	3.2%
**Detailed Workplan and Deliverables Schedule**	5%	The completion schedule for each deliverable is realistic.	3.8%
**Leadership and Organizational Capability**	Yes/No	Are the attributes of the requesting institution, organization, or association appropriate and sufficient to support and facilitate the execution of the project?	Yes
**Staff Capacity**	Yes/No	Is there sufficient evidence that the applicants have the staff expertise, experience and resources to properly conduct this project?	Yes
**Budget**	Yes/No	Does the total amount of money requested seem appropriate in relation to the project objectives and methodology?	Yes
		**Overall Score** (Max 100%)	74.6%

	Maximum Score	Evaluation Criteria Guidelines	Avg. Reviewer Score for this Proposal (%)
		**Overall Rating** (Grade scale independent of score)	"B+"
		**Overall Summary Recommendation:**	ACCEPT

Strengths:

-Strengths of this proposal include a focus on an important and understudied aspect of chronic pain for breast cancer providers.
-The literature review is complete and sophisticated. There is an adequate evidence base for the proposed intervention.
-The study design and methods are appropriate and sophisticated. The expertise and capacity of this team to conduct a high-quality study are clearly apparent.
-The comparison group is current standard care for the targeted condition
-Precise inclusion and exclusion criteria are provided.
-There is a very solid study protocol including enrollment, randomization, data collection, etc.
-Power calculations and statistical analysis plan is excellent. Approach to missing data is solid.
-Excellent strategies to promote ongoing patient engagement and retention.

Weaknesses:

-It is stated that "successful completion of the purposed project will directly benefit all women... " However, providing an intervention on the Internet makes it equally available to all women who have survived breast cancer. There are many barriers that might prevent women from disadvantaged populations from gaining similar access and benefit as more affluent women.
-The language used to describe the results of the pilot testing is informal. More precise quantitative and qualitative descriptions would have been helpful.
-It is too ambitious to assume that an educational intervention delivered by internet will produce a significant change in weight.

-unclear how co-variates will be controlled for
--do not control for neuropathic pain and medication treatment of pain [it's very possible that a large percentage of the pain with these women may not be lymph flow dependent but nerve injury dependent]

Overall Comments:

This is perhaps the best proposal I have read during my tenure on this review panel. The methodological weaknesses described above are only minor.
